# Thermal prediction of turbulent forced convection of nanofluid using computational fluid dynamics coupled genetic algorithm with fuzzy interface system

**DOI:** 10.1038/s41598-020-80207-2

**Published:** 2021-01-14

**Authors:** Meisam Babanezhad, Iman Behroyan, Ali Taghvaie Nakhjiri, Mashallah Rezakazemi, Azam Marjani, Saeed Shirazian

**Affiliations:** 1 Institute of Research and Development, Duy Tan University, 550000 Da Nang, Vietnam; 2 Faculty of Electrical-Electronic Engineering, Duy Tan University, 550000 Da Nang, Vietnam; 3 Department of Artificial Intelligence, Shunderman Industrial Strategy Co., Tehran, Iran; 4 Faculty of Mechanical and Energy Engineering, Shahid Beheshti University, Tehran, Iran; 5 Department of Computational Fluid Dynamics, Shunderman Industrial Strategy Co., Tehran, Iran; 6 Department of Petroleum and Chemical Engineering, Science and Research Branch, Islamic Azad University, Tehran, Iran; 7 Faculty of Chemical and Materials Engineering, Shahrood University of Technology, Shahrood, Iran; 8 Department for Management of Science and Technology Development, Ton Duc Thang University, Ho Chi Minh City, Vietnam; 9 Faculty of Applied Sciences, Ton Duc Thang University, Ho Chi Minh City, Vietnam; 10 Laboratory of Computational Modeling of Drugs, South Ural State University, 76 Lenin prospekt, 454080 Chelyabinsk, Russia

**Keywords:** Engineering, Mathematics and computing

## Abstract

Computational fluid dynamics (CFD) simulating is a useful methodology for reduction of experiments and their associated costs. Although the CFD could predict all hydro-thermal parameters of fluid flows, the connections between such parameters with each other are impossible using this approach. Machine learning by the artificial intelligence (AI) algorithm has already shown the ability to intelligently record engineering data. However, there are no studies available to deeply investigate the implicit connections between the variables resulted from the CFD. The present investigation tries to conduct cooperation between the mechanistic CFD and the artificial algorithm. The genetic algorithm is combined with the fuzzy interface system (GAFIS). Turbulent forced convection of Al_2_O_3_/water nanofluid in a heated tube is simulated for inlet temperatures (i.e., 305, 310, 315, and 320 K). GAFIS learns nodes coordinates of the fluid, the inlet temperatures, and turbulent kinetic energy (TKE) as inputs. The fluid temperature is learned as output. The number of inputs, population size, and the component are checked for the best intelligence. Finally, at the best intelligence, a formula is developed to make a relationship between the output (i.e. nanofluid temperatures) and inputs (the coordinates of the nodes of the nanofluid, inlet temperature, and TKE). The results revealed that the GAFIS intelligence reaches the highest level when the input number, the population size, and the exponent are 5, 30, and 3, respectively. Adding the turbulent kinetic energy as the fifth input, the regression value increases from 0.95 to 0.98. This means that by considering the turbulent kinetic energy the GAFIS reaches a higher level of intelligence by distinguishing the more difference between the learned data. The CFD and GAFIS predicted the same values of the nanofluid temperature.

## Introduction

Recently, an attempt has been made to improve the efficiency of heat transfer fluids by adding inert solid particles (e.g. alumina) to the fluid for heat transfer applications. If the added particles are at nano size, the fluid can be recognized as nanofluid. These nanofluids are novel heat transfer fluids that are made by dispersion of particles in nanometer sizes in a base working fluid such as refrigerants, oil, water, etc. Utilizing the metallic nanoparticle suspension (such as copper, silver, silicon, and aluminum) has shown more augmentation of the nanofluid conductivity than their conventional base fluids^1–4^. So, the nanofluids have made a promising area for increasing the heat transfer efficiency in many kinds of applications. Comparing micro- and milli- sized particles, the suspension of the nanoparticles could minimize the drawback of clogging^5^.

The mentioned advantages of the nanofluids as working fluids took the attention of researchers and as a result, a progressive trend has been made in heat transfer investigations for nanofluids both experimentally and computationally as well. The numerical study conducted in^6^ deals with assessing the thermal and hydrodynamic behaviors of completely established turbulent flow of alumina + water nanofluids. Behroyan et al.^7,8^ analyzed the importance of the single and two-phase modeling on the numerical investigations of the nanofluid convective flows. They reported that the single-phase model could be considered for modeling the nanofluid flow if the thermos-physical properties of the nanofluid are adopted precisely. Bahmani et al. ^9^ have modeled the heat transfer of the turbulent flow of a nanofluid in a heat exchanger. The increase of the outlet and the wall temperatures by the nanoparticle volume fraction was reported in their study. Bianco et al.^10^ numerically simulated and reported the laminar convection of the alumina water based nanofluid in a channel with a rectangular cross-section. It was reported that using the nanofluid, the wall of the channel is cooler than that of using the base fluid. However, the nanofluids impose more pumping power as a result of the more pressure drop than water. Benkhedda et al.^11^ reported the influence of solid particles shape on heat transfer efficiency as well as pressure loss. According to the results, the most rate of heat transfer was related to the nanoparticle in the blade shape, while the maximum pressure drop is for the shape of the platelet. Sharma et al.^12^ highlighted the importance of the nanoparticle material and the base fluid properties on heat transfer efficiency. Zainon and Azmi^13^ reported the heat transfer improvement for the suspension of the hybrid nanoparticles in the mixture of water and Bio-Glycol.

Computational fluid dynamics (CFD) is known as a precise and reputable approach in engineering design and troubleshooting purposes. The predicted thermal and hydrodynamic parameters of fluid flows could help engineers and researchers in the trial and error expenses of the experiments. Although the CFD could predict all variables of the fluid flow, the connections between such variables with each other could not be found by this approach. There are some fluid flow variables related to each other implicitly and the mathematical equations or functions for connecting them cannot be achieved easily. In this case, the machine learning technique of the artificial intelligence (AI) algorithm is a way for finding the data patterns and their changes. The AI algorithms cannot reach the maximum intelligence level (the best intelligence) unless the connections of all learned data are found from each other, and the optimum structure needs to be specified. Although the AI algorithms have been used for data analysis for years in different concepts^14–21^, a few studies have recently shown the application of the artificial intelligence algorithms in simplification of the CFD modeling^22–29^. The critical literature review on numerical simulating nanofluid flows shows a deep gap in the applications of artificial intelligence in combination of CFD modeling to establish hybrid simulation methodology. The most studies are simple presentations of the AI algorithm of the adaptive network-based fuzzy interface system (ANFIS) for CFD data capture dealing with fluid flow and transport phenomena^30^.

For addressing such a research gap, this study reports a novle methodology on the CFD simulating of nanofluid flowing in a heated pipe at turbulent regime. The genetic algorithm based fuzzy interface system, known as GAFIS, is used to learn the CFD results. This investigation tries to develop a correlation relating the temperature distribution of the nanofluid flow to fluid flow nodes position, the inlet temperature, and the turbulent kinetic energy using the GAFIS artificial intelligence. This approach would be a sample idea for finding the implicit function between the fluid flow variables.

## Methodology

### CFD approach

In the current work, a cylindrical tube is taken into account in a horizontal position (L = 1 m and DI = 0.01 m). Constant heat flux (85 kW/m^2^) is used for the tube wall. The inlet velocity of the nanofluid is 0.91 m/s, while the inlet temperature changes by different values of 300, 305, 310, 315, and 320 K. The main equations used here are^7,31,32^:

Continuity equation:

1 $$\nabla .\left({\rho }_{eff}\overrightarrow{U}\right)=0.$$

Momentum equation:

2 $$\nabla .\left({\rho }_{eff}\overrightarrow{U}\overrightarrow{U}\right)=-\nabla P+\nabla .({\mu }_{eff}\nabla \overrightarrow{U}-{\rho }_{eff}\overline{UU)}.$$

Energy equation:

3 $$\nabla .\left({\rho }_{eff}{C}_{p.eff}\overrightarrow{U}T\right)=\nabla .\left(({k}_{eff}+{k}_{turb})\nabla T\right).$$

The relevant equations for the $k-\varepsilon$ turbulence model is written as ^7,31,33,34,30^:

4 $$\nabla .(\rho_{eff} KU) = \nabla .[(\frac{{\mu_{t} }}{{\sigma_{k} }})\nabla (K)] + G_{k} - \rho_{eff} \varepsilon ,$$

5 $$\nabla .(\rho_{eff} \varepsilon U) = \nabla .[\frac{{\mu_{turb} }}{{\sigma_{\varepsilon } }}\nabla \varepsilon ] + \frac{\varepsilon }{k}(C_{1\varepsilon } G_{k} - C_{2\varepsilon } \rho_{eff} \varepsilon ),$$

6 $$G_{k} = \mu_{turb} (\nabla U + (\nabla U)^{turb} ), \, \mu_{turb} = \rho_{eff} C_{\mu } \frac{{K^{2} }}{\varepsilon } ,$$

$$C_{\mu } = 0.09,\sigma_{k} = 1.00,\sigma_{\varepsilon } = 1.30,C_{1\varepsilon } = 1.44,C_{2\varepsilon } = 1.92$$

The temperature-dependent correlations for water properties are given as follows^35,29^:

Density ^36^:

7 $$\rho_{water} = 2446 - 20.674T + 0.11576T^{2} - 3.12895 \times 10^{ - 4} T^{3} + 4.0505 \times 10^{ - 7} T^{4} - 2.0546 \times 10^{ - 10} T^{5},$$

Viscosity ^37^:

8 $$\mu_{water} = \alpha 10^{{\left( {\frac{\beta }{T - \delta }} \right)}},$$

where, α = 2.414 × 10^–5^, β = 247.8, and δ = 140.

Specific heat ^36^:

9 $$\left( {C_{p} } \right)_{water} = \exp \left( {\frac{8.29041 - 0.012557T}{{1 - \left( {1.52373 \times 10^{ - 3} } \right)T}}} \right) .$$

The properties of the nanofluid are estimated using ^35–37^:

10 $${\rho }_{eff}=\left(1-\varnothing \right){\rho }_{water}+\varnothing {\rho }_{s},$$

11 $${c}_{p,eff}=\frac{(1-\varnothing )(\rho {c}_{p}{)}_{water}+\varnothing (\rho {c}_{p}{)}_{s}}{(1-\varnothing ){\rho }_{water}+\varnothing {\rho }_{s}},$$

For the nanofluid viscosity, the correlation suggested by Maiga et al.^6^ is employed.

12 $$\mu_{eff} = (1 + 7.3\phi + 123\phi^{2} )\mu_{water},$$

The thermal conductivity could be also obtained as Chon et al. ^37^ recommended:

13 $$k_{eff} /k_{water} = 1 + 64.7\left( \phi \right)^{0.7460} \left( {\frac{{d_{water} }}{{d_{s} }}} \right)^{0.3690} \left( {\frac{{k_{water} }}{{k_{s} }}} \right)^{0.7476} Pr^{0.9955} Re_{np}^{1.2321},$$

$$\left\{\begin{array}{c}294<T<344\\ 11nm<ds<150nm,\end{array}\right.,$$

14 $$Re_{np} = \frac{{\rho_{water} B T}}{{3\pi \mu_{water}^{2} \gamma }},$$

where γ = 0.17 nm is defined as the mean free path between water molecules and B is the constant of Boltzmann. This correlation is recommended for Al_2_O_3_/water nanofluid and the temperature of 294 K to 344 K.

### Grid dependency test

The meshing process was carried out using the design modeller tool existing in ANSYS. Two different mesh densities were selected for testing the independence of the models from the mesh size (i.e. 1,074,537 nodes and 6,751,125 nodes). The temperatures obtaining from both mesh sizes were the same. So, all CFD simulations have been done based on the first mesh density.

### CFD validation

For verification of the CFD results, the average Nusselt number of the dilute Al_2_O_3_/water nanofluid (i.e. 0.08 volume fraction) of this simulation is compared with the measured results taken from the literature. Table 1 illustrates the sample results of both studies with an acceptable agreement.

**Table 1:** CFD verification test.

	Study condition	Nu
Present study	Re = 10.846 × 10^3^ 0.08 vol.% Al_2_O_3_/water	89.37
Experimental study^38^	Re = 9.65 × 10^3^ 0.135 vol.% Al_2_O_3_/water	74.10
	Re = 10.90 × 10^3^ 0.067 vol.% Al_2_O_3_/water	84.85

### GAS (genetic algorithms)

Essentially, genetic algorithms are for searching in terms of natural genetics and nature’s mechanics (such as natural selection, the fittest survival)^39,40^. Indeed, GAS analyzes the encodings of the parameters rather than the real ones. These factor sets are decreased to some signs from an arbitrary, yet operative alphabet and the solutions are assessed in terms of the given symbol structures. A population of solution structures is maintained by GAS through the procedure; hence, they are not restricted by selecting the primary single point solution guesses. Only the encoding techniques and objective function should be defined within the programmer in the case of GAS^39–41^.

### Fuzzy inference system (FIS)

FIS is based on training process, and is capable to utilize other algorithms as a trainer for example using ant colont optimization algorithm called ACOFIS and using artificial neural network as a trainer called ANFIS^30^. There are three types of fuzzy sets proposed and implemneted by Takagi and Sugeno ^42^ where the rth rule function is stated as ^42,43^:

15 $${w}_{r}={\mu }_{Ar}\left({\theta}\right) {\mu }_{Br}\left(r\right){\mu }_{Cr}(z) {\mu }_{Dr}(Tin){\mu }_{Er}(TKE),$$

where ${w}_{r}$ represents the weight of each rule in the fuzzy structure, and $\mu$ indicates the membership functions (MF) incoming signals based on each inputs, e.g. angle (θ), radius (r), z-direction (z), inlet temperature (T_in_), and turbulence kinetic energy (TKE) in this work.

The amount of firing strength is determined for each rule^42,44^:

16 $$\stackrel{-}{{w}_{r}}=\frac{{w}_{r}}{\sum \left({w}_{r}\right)},$$

where $\stackrel{-}{{w}_{r}}$ is known as normalized firing strengths. The function of a consequence if–then rule presented by Takagi and Sugeno is applied ^42^.

Consequently, the function of the nodes is ^42,45,29^:

17 $$\stackrel{-}{{w}_{r}}{f}_{r}=\stackrel{-}{{w}_{r}}({o}_{r}{\theta}+{p}_{r}r+{q}_{r}z+{r}_{r}Tin+{s}_{r}TKE+{u}_{r}),$$

where ${{o}_{r} , {p}_{r} , q}_{r}$, ${r}_{r}$, ${s}_{r}$_*,*_ and ${u}_{r}$ show the parameters of the if–then rules and are termed consequent parameters. We have developed and implemented this approach in our previous works for simulation of physical systems^21,24,45–49,29^.

## Results and discussion

**Figure 1:**
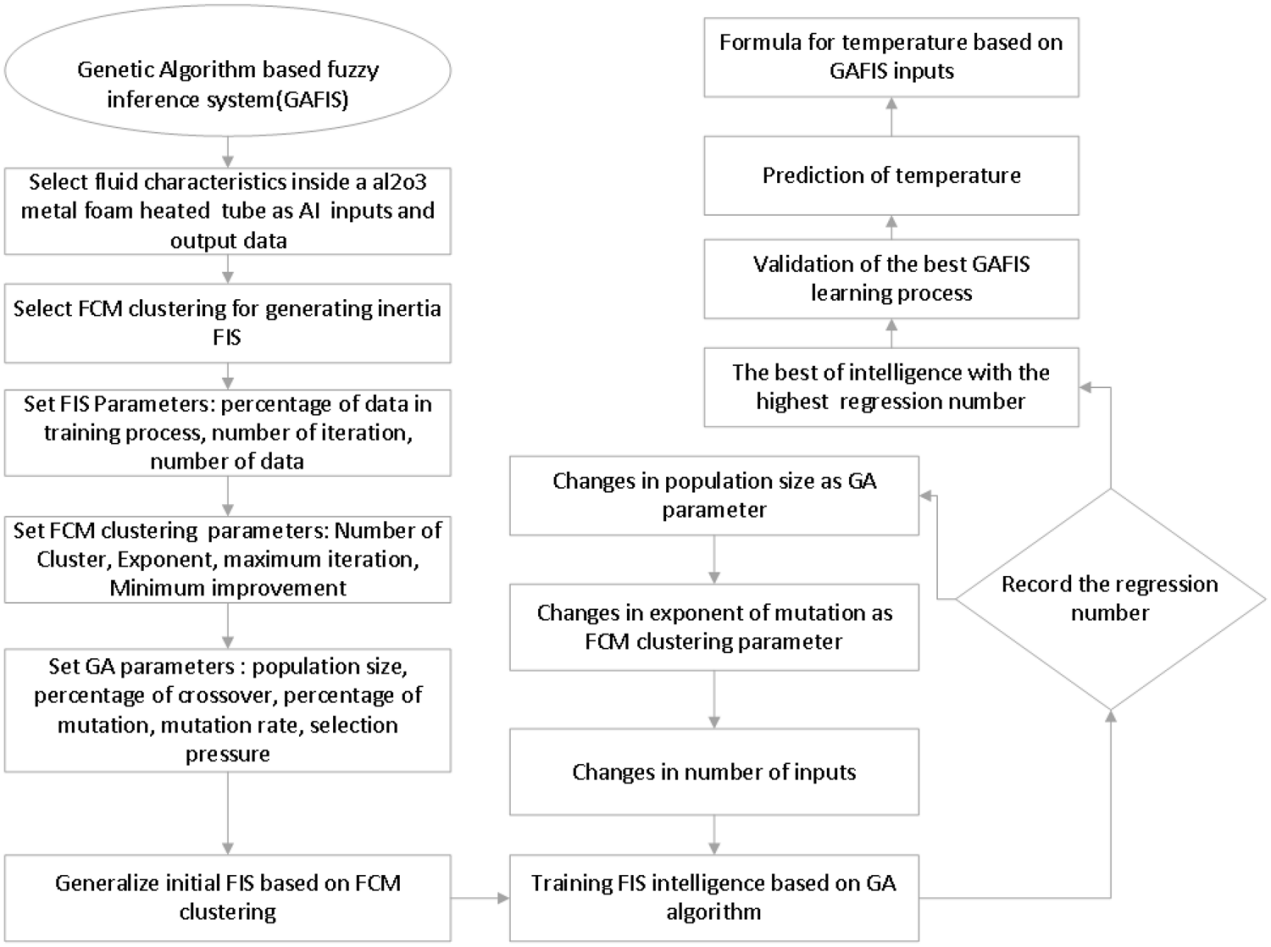
Flowchart of GAFIS method.

After finding the intelligence, the implicit function between the flow variables could be developed. This study is intended to show how the genetic algorithm with a fuzzy interface system (GAFIS) can be used for this purpose. The turbulent nanofluid flow of Al_2_O_3_/water in a circular tube is the case for CFD modeling. The single-phase theory (i.e. homogeneous distribution of nanoparticles in the based fluid) is considered for the nanofluid modeling. Figure 1 illustrates all steps for setup and prediction of the target variables of the GAFIS. The GAFIS trains the inlet temperature, the cylindrical coordinates (i.e. r, ϴ, and z) of the nodes in the pipe domain and their corresponding turbulent kinetic energy (TKE), as inputs, and the temperature of the nodes, as the output. The number of inputs increases step by step until the best intelligence condition is met^32^.

The fuzzy C-means clustering (FCM) is selected for generating inertia FIS in this simulation case study^29^. For setting FIS parameters, the number of data, the number of iterations, and the percentage of data in the training are determined equal to 24,164, 150, and 75% respectively. As the FCM parameter, 10 clusters are considered for each input. Different numbers of exponents (i.e. 2, 3, 4, and 5), as the FCM parameter, are checked during the modeling so that the best intelligence is derived. The population size as one of the genetic algorithm parameters are also checked for the values of 5, 10, 20, and 30 until the best intelligence is found. After the definition of all parameters, a genetic algorithm based on FIS begins the training of the data. The temperature of the nanofluid predicted by the GAFIS is compared by that predicted by the CFD. The more regression number, the best intelligence is achieved.

**Figure 2:**
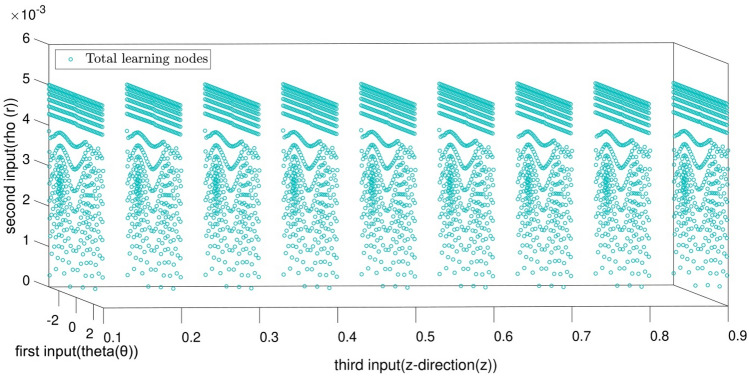
Learning data which is CFD output.

**Figure 3:**
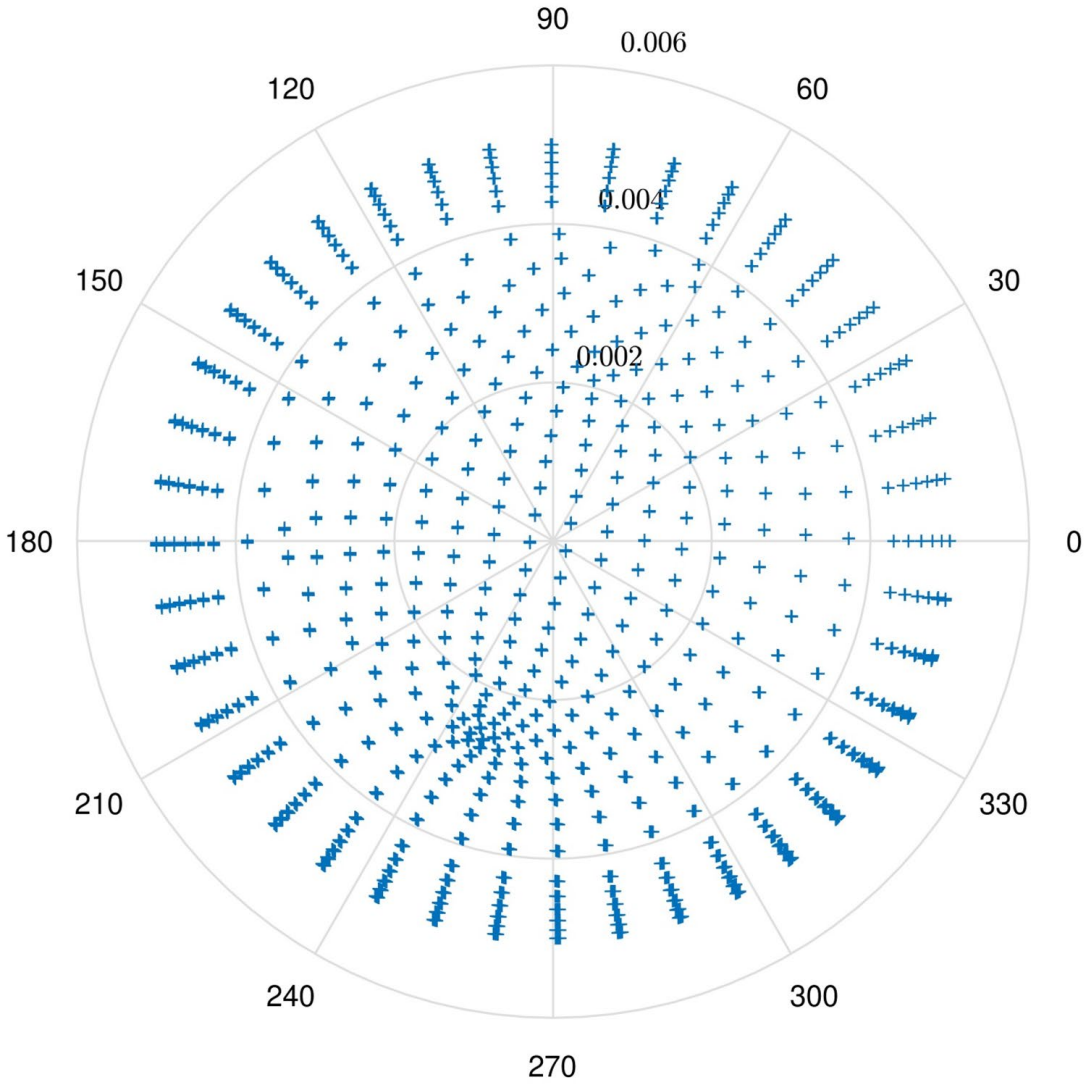
The cross-section of learning nodes.

Figure 2 shows the domain of the nodes learned by the GAFIS. According to Fig. 2, the r is between 0 to 5(mm), the ϴ is between ± 2 (rad), and the z is between 0.1 to 0.9 (m). The cross-section view of the nodes is also shown in Fig. 3.

**Figure 4:**
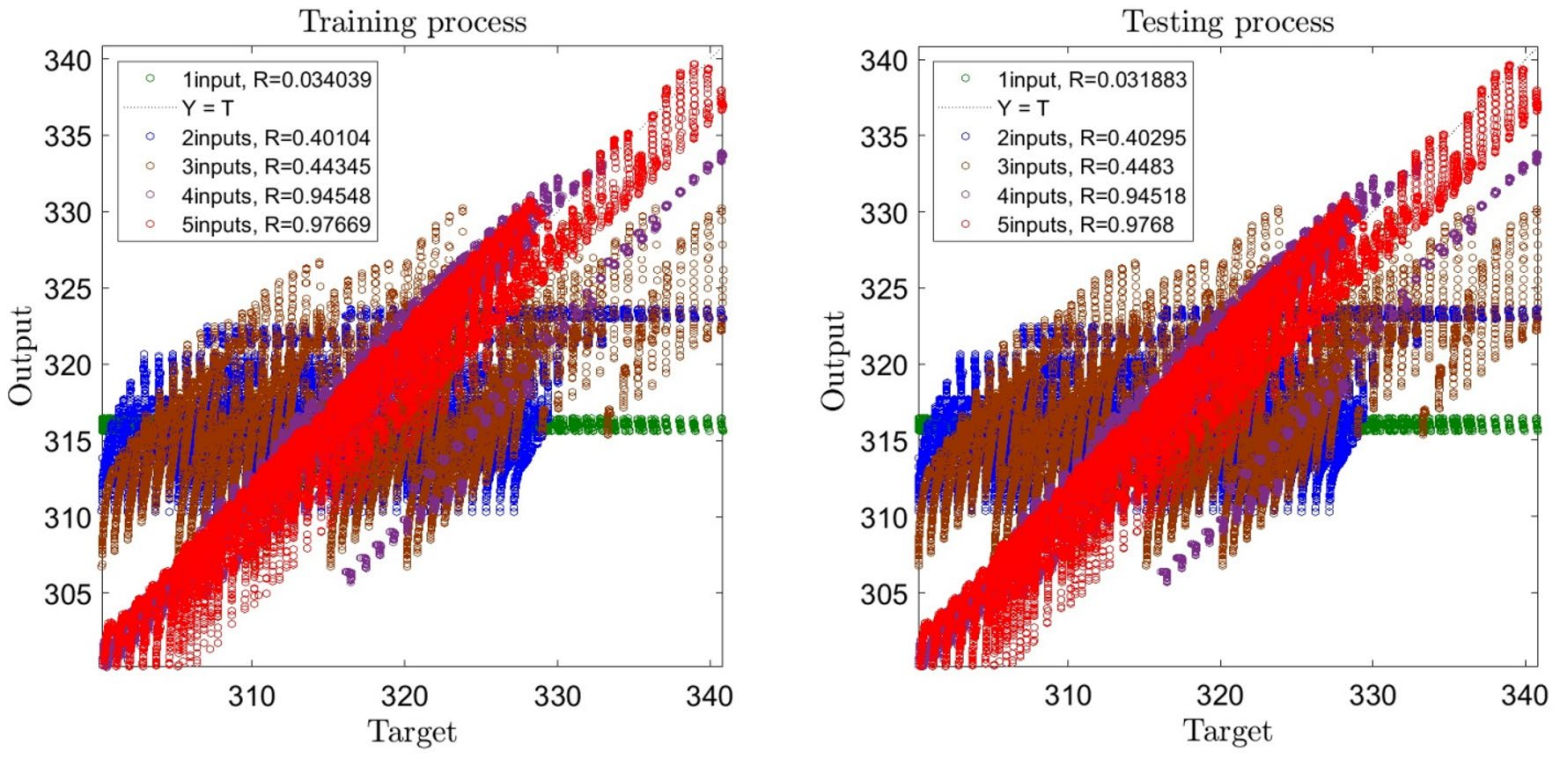
Learning process with changes in number of inputs from one to five.

Figure 4 shows the regression number changes by adding the inputs. The highest regression number (i.e. 0.97) is obtained by considering all 5 inputs (i.e. r, θ, z, inlet temperature, and TKE). It should be noted that by adding the turbulent kinetic energy as the fifth input the regression value increases from 0.95 to 0.98. This means that considering the turbulent kinetic energy the GAFIS reaches a higher level of intelligence by distinguishing the more difference between the learned data.

**Figure 5:**
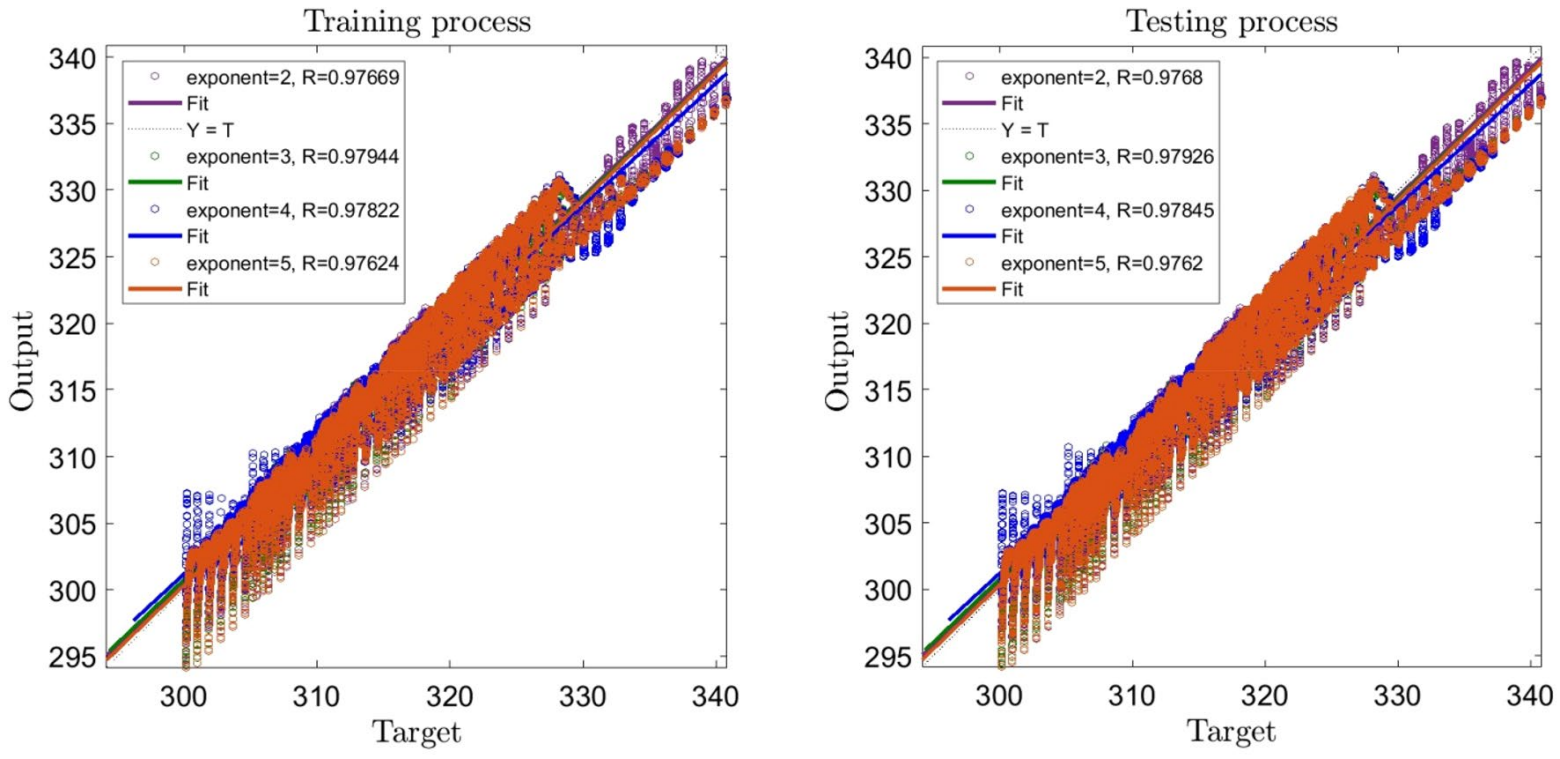
Learning process with changes in exponent as FCM clustering parameter (2,3,4,5).

**Figure 6:**
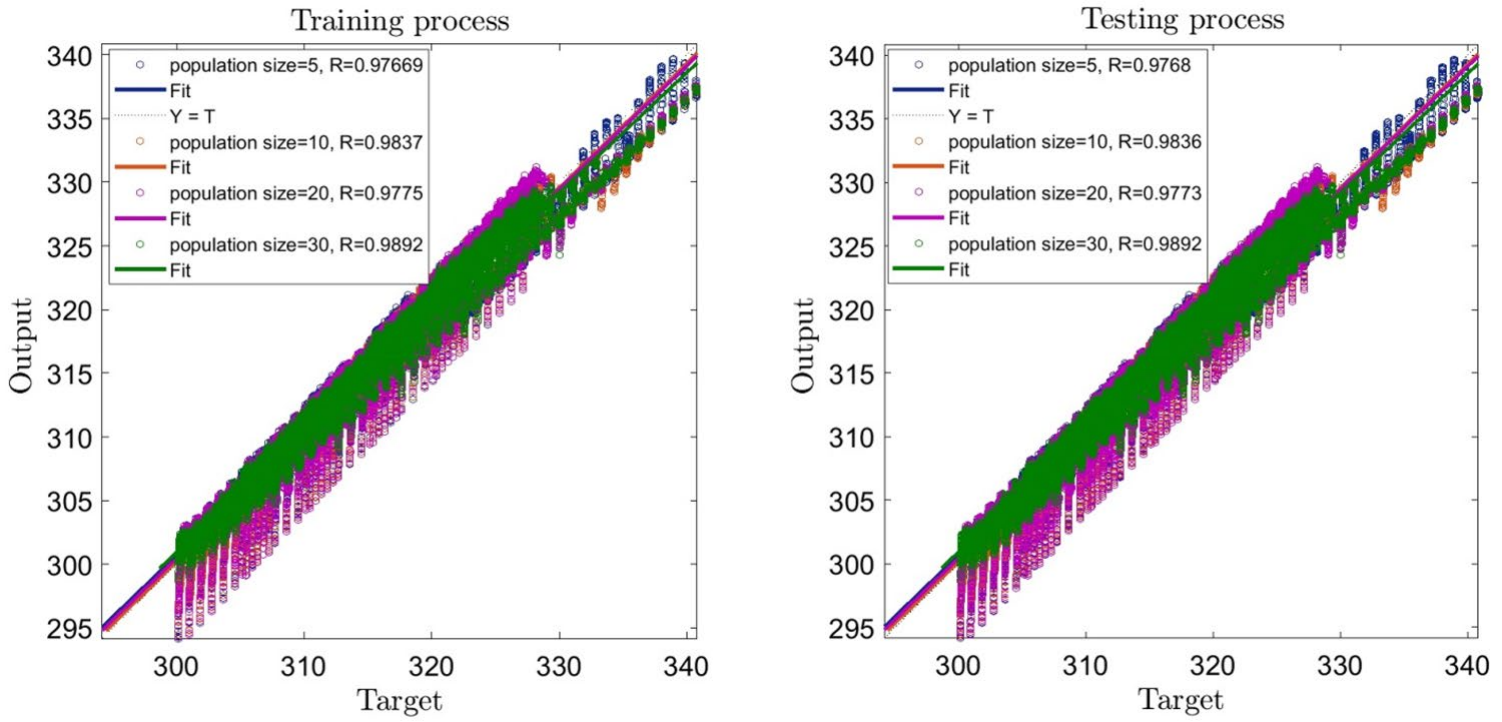
Learning process with changes in population size as GA parameter (5,10,20,30).

According to Figs. 5 and 6, the highest regression number (i.e. 0.979) is related to the exponent of 3 and the population size of 30. So, the best intelligence can be found for 5 inputs, the exponent of 3, and the population size of 30.

**Figure 7:**
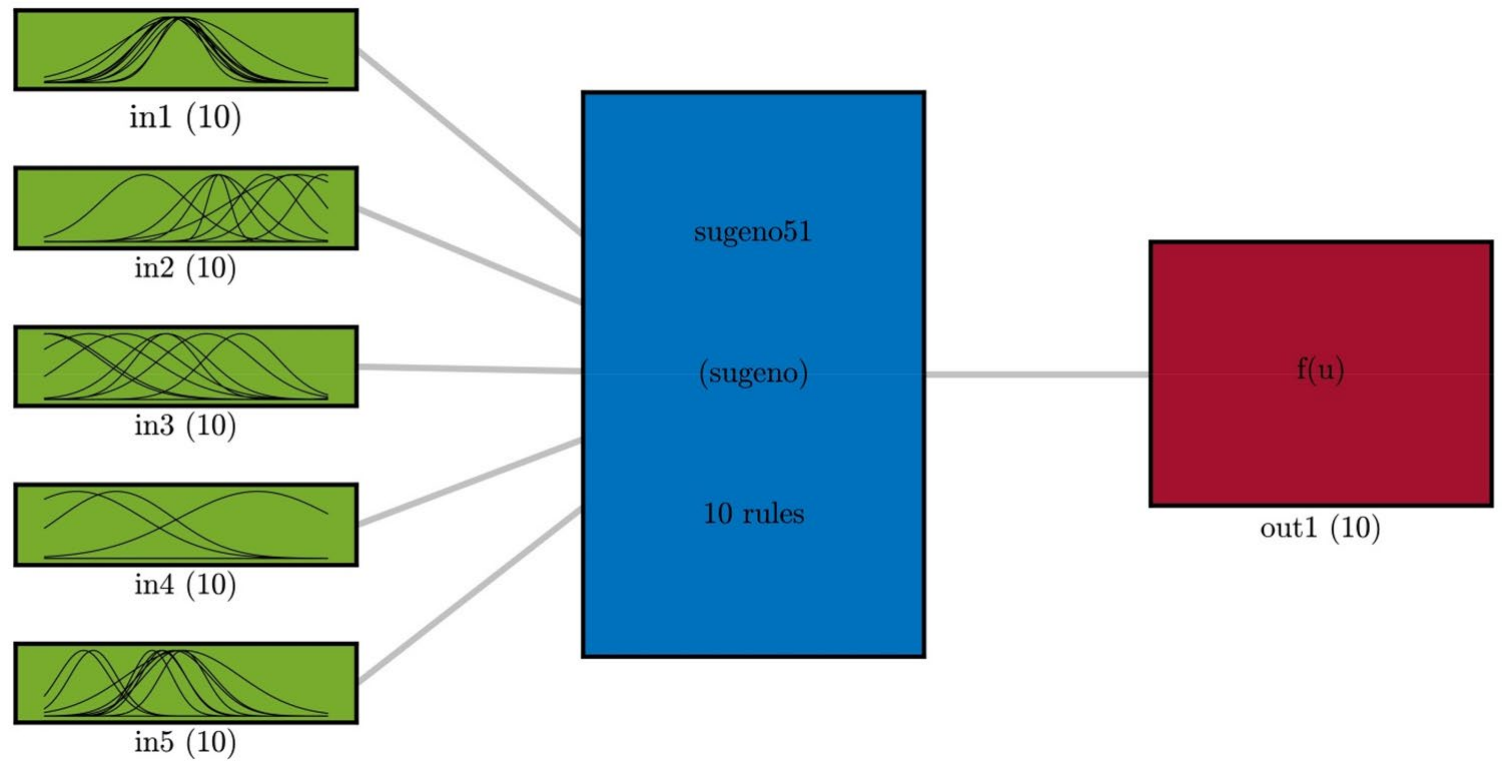
GAFIS structure in the best result of learning.

**Figure 8:**
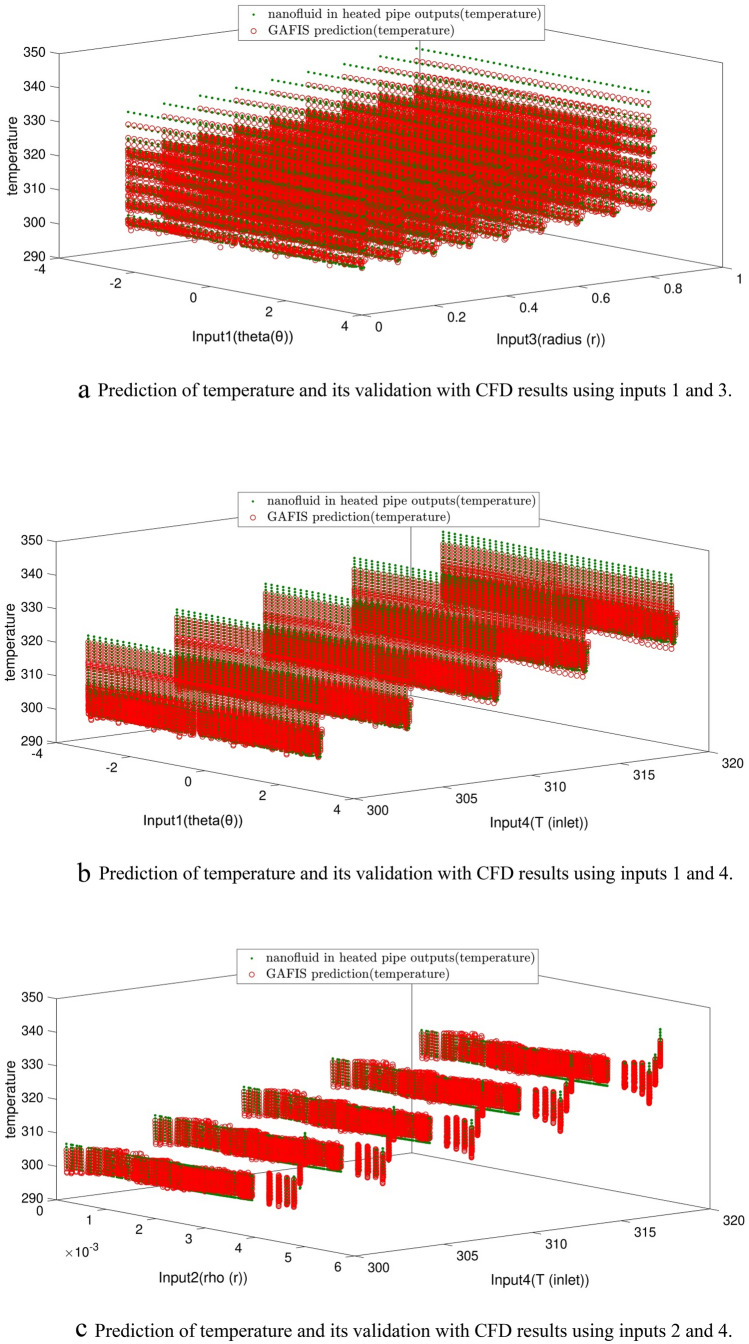
**(a)** Prediction of temperature and its validation with CFD results using inputs 1 and 3. **(b)** Prediction of temperature and its validation with CFD results using inputs 1 and 4. **(c)** Prediction of temperature and its validation with CFD results using inputs 2 and 4.

**Figure 9:**
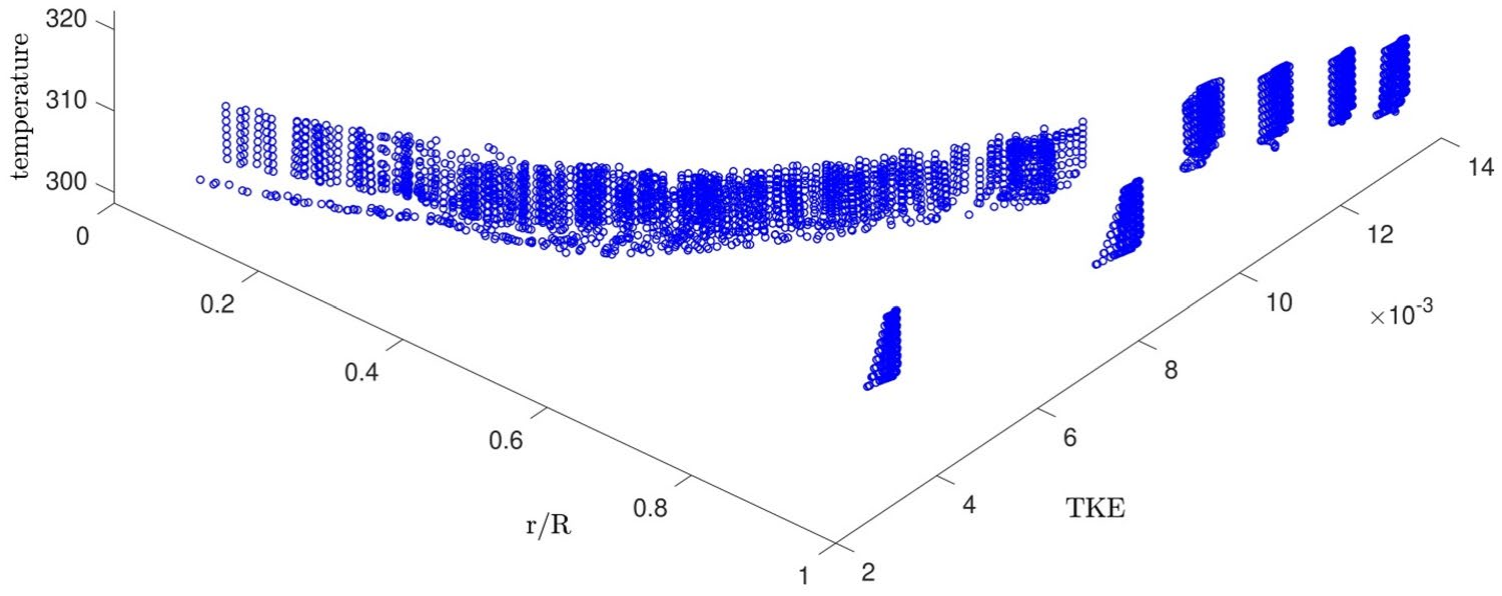
The temperature distribution for different inlet temperatures versus the turbulent kinetic energy and the dimensionless positions of the nodes.

Schematic diagram of the GAFIS structure is shown in Fig. 7. The number cluster for each input, the number of rules, and the number of membership functions of the output all are equal to 10. The Gaussian function is considered for membership function in this case. The shape of the Gaussian function is schematically shown on the left side of Fig. 7. The temperatures of the CFD prediction are compared with those of the GAFIS. A high compatibility exists between both predictions as depicted in Fig. 8. Figure 9 illustrates the temperature distribution of the nodes for different inlet temperatures versus the turbulent kinetic energy and the dimensionless positions of the nodes. As expected, the TKE is zero on the wall. This is because there is not any slip velocity on the wall. The TKE is maximum in the vicinity of the wall and within the hydrodynamic boundary layer. As getting closer to the pipe centerline and far from the wall, the TKE decreases.

Finally, a general formula is developed to determine the nanofluid temperature in domain depending on the inputs. According to Eq. (18), once the optimum intelligence of the model is established, the consequent factors and the parameters of the Gaussian function can be found^32^. Table 2 shows the general equation of the Gaussian function and its parameters (i.e. C, and σ). Table 3 indicates the Gaussian function parameters for each cluster in each input. So, there are 10 sets of data, based on 10 clusters, for each of 5 inputs. Table 4 also shows 10 sets of the consequent parameters (i.e. o_m_ , p_m_, q_m_, r_m_, s_m_, and u_m_) for all 10 clusters. In this way, the temperature of the nanofluid can be found relating to any values of the inlet temperature and anywhere in the domain without CFD modeling.

**Table 2:** Gaussian membership function equation in GAFIS learning process.

Membership function	Equation
Gaussian	${e}^{\frac{-{\left(x-c\right)}^{2}}{2{\sigma }^{2}}}$

**Table 3:** Inputs membership functions parameters in GAFIS intelligence learning process.

Number of cluster	Type of MFs	σ	C		Number of cluster	Type of MFs	σ	C
'in1cluster1'	'gaussmf'	0.826635429	− 0.257424169		'in3cluster6'	'gaussmf'	0.15393271	0.106704257
'in1cluster2'	'gaussmf'	0.576086004	− 0.028883971		'in3cluster7'	'gaussmf'	0.111842544	− 0.858703931
'in1cluster3'	'gaussmf'	0.757543057	− 0.263228836		'in3cluster8'	'gaussmf'	0.174190572	0.229131334
'in1cluster4'	'gaussmf'	0.69040043	− 0.206462468		'in3cluster9'	'gaussmf'	0.105266664	0.655767376
'in1cluster5'	'gaussmf'	0.85294523	− 0.239048447		'in3cluster10'	'gaussmf'	0.084140256	1.133442934
'in1cluster6'	'gaussmf'	0.730274295	− 0.354283281		'in4cluster1'	'gaussmf'	1.618098253	379.5749903
'in1cluster7'	'gaussmf'	1.358895591	− 0.103305891		'in4cluster2'	'gaussmf'	4.009478411	305.0873344
'in1cluster8'	'gaussmf'	0.958817478	− 0.414412627		'in4cluster3'	'gaussmf'	3.707930813	112.4063766
'in1cluster9'	'gaussmf'	0.501674052	− 0.122800932		'in4cluster4'	'gaussmf'	5.965432157	160.8270081
'in1cluster10'	'gaussmf'	− 0.760020363	− 0.017918213		'in4cluster5'	'gaussmf'	4.629180724	302.3176448
'in2cluster1'	'gaussmf'	0.000642441	0.003135137		'in4cluster6'	'gaussmf'	2.530182626	89.77626929
'in2cluster2'	'gaussmf'	0.000509462	0.003960464		'in4cluster7'	'gaussmf'	9.846234379	375.0116229
'in2cluster3'	'gaussmf'	0.000532355	0.004366219		'in4cluster8'	'gaussmf'	5.479436302	315.0356584
'in2cluster4'	'gaussmf'	− 0.000434711	0.003151819		'in4cluster9'	'gaussmf'	2.951138918	333.0374101
'in2cluster5'	'gaussmf'	0.000668226	− 0.001951237		'in4cluster10'	'gaussmf'	2.872943793	250.0895913
'in2cluster6'	'gaussmf'	0.001099873	0.004459562		'in5cluster1'	'gaussmf'	0.001163133	0.00398334
'in2cluster7'	'gaussmf'	0.000731798	0.001894067		'in5cluster2'	'gaussmf'	0.001066247	0.004421655
'in2cluster8'	'gaussmf'	− 0.000194298	0.003152282		'in5cluster3'	'gaussmf'	0.00103551	0.007600883
'in2cluster9'	'gaussmf'	7.53E− 05	− 0.000877803		'in5cluster4'	'gaussmf'	0.000487411	0.025364583
'in2cluster10'	'gaussmf'	0.000465181	0.004910869		'in5cluster5'	'gaussmf'	0.001637584	0.008089513
'in3cluster1'	'gaussmf'	0.154370243	0.324098633		'in5cluster6'	'gaussmf'	0.001580118	0.008324729
'in3cluster2'	'gaussmf'	0.08881652	0.443553946		'in5cluster7'	'gaussmf'	0.001041173	0.00849897
'in3cluster3'	'gaussmf'	0.133380146	0.558057507		'in5cluster8'	'gaussmf'	0.000919726	0.007106961
'in3cluster4'	'gaussmf'	− 0.119917998	0.44296301		'in5cluster9'	'gaussmf'	0.002619051	0.008503345
'in3cluster5'	'gaussmf'	0.158536818	0.116304464		'in5cluster10'	'gaussmf'	0.001112753	0.007515792

**Table 4:** GAFIS method consequent parameters for predicting temperature.

	Output MFs	Output MFs type	o	p	q	r	s	u
1	'out1cluster1'	'linear'	0.078996019	2357.527744	10.09732893	0.947178031	− 778.9879676	9.56412065
2	'out1cluster2'	'linear'	0.079820308	4281.41413	9.180047255	0.954597364	− 1116.136518	9.56412065
3	'out1cluster3'	'linear'	0.057378996	4585.748672	8.884517906	0.233957643	− 1114.194728	2.684024609
4	'out1cluster4'	'linear'	0.107061914	3411.50903	10.92249369	1.412137104	− 1114.194728	5.112260621
5	'out1cluster5'	'linear'	0.080157462	3014.783586	9.180077769	0.954597364	− 1116.270466	14.27288647
6	'out1cluster6'	'linear'	0.080157462	4181.356389	11.6245228	0.605227692	− 911.4273206	10.47376635
7	'out1cluster7'	'linear'	0.080157462	4556.321232	7.710165876	0.950779527	− 806.6211222	22.62104405
8	'out1cluster8'	'linear'	2.31E− 01	4191.570094	7.462891624	0.954597364	− 1171.080269	9.56412065
9	'out1cluster9'	'linear'	0.082528737	4190.388858	8.902586343	0.954597364	− 917.8175631	9.56412065
10	'out1cluster10'	'linear'	0.07993579	2582.906758	7.853218312	− 0.42716382	− 1261.150587	9.562570627

18 $$temperature=\frac{\sum_{i=1}^{10}\sum_{j=1}^{10}\sum_{k=1}^{10}\sum_{l=1}^{10}\sum_{n=1}^{10}\left({\mu }_{1i}\times {\mu }_{2j}\times {\mu }_{3k}\times {\mu }_{4l}\times {\mu }_{5n}\right)\times \left({o}_{m}\theta \times {p}_{m}r\times {q}_{m}z\times {r}_{m}{T}_{inlet}\times {s}_{m}TKE\times {u}_{m}\right)}{\sum_{i=1}^{10}\sum_{j=1}^{10}\sum_{k=1}^{10}\sum_{l=1}^{10}\sum_{n=1}^{10}\left({\mu }_{1i}\times {\mu }_{2j}\times {\mu }_{3k}\times {\mu }_{4l}\times {\mu }_{5n}\right)},$$

In which:

19 $$\mu_{1i} = e\frac{{ - (x - c_{j} )^{2} }}{{(2\sigma^{2} )}},\mu_{2j} = e\frac{{ - (x - c_{j} )^{2} }}{{(2\sigma^{2} )}},\mu_{3k} = e\frac{{ - (x - c_{j} )^{2} }}{{(2\sigma^{2} )}},\mu_{4l} = e\frac{{ - (x - c_{j} )^{2} }}{{(2\sigma^{2} )}}\quad {\text{and}}\;\mu_{5n} = e\frac{{ - (x - c_{j} )^{2} }}{{(2\sigma^{2} )}},$$

## Conclusion

This work was aimed to provide a facile approach to connect the fluid flow characteristics resulted from the computational fluid dynamic (CFD) predictions. The CFD is able to predict all hydro-thermal parameters of fluid flows. But there is no way to find the connections such parameters with each other using the CFD. Machine learning by the artificial intelligence (AI) algorithm could intelligently record the data patterns. However, such a pattern record does not exist for the CFD data. For this purpose, the simulation of convection of Al_2_O_3_/water nanofluid in a tube was simulated at the turbulence flow regime. The artificial intelligence of the genetic algorithm with the fuzzy interface system was used for integration with the CFD. The cylindrical coordinates (i.e. r, ϴ, and z) of the CFD nodes, the inlet temperature, and the turbulent kinetic energy (TKE) were learned as inputs to predict the temperature of the nodes in the system of interest. Different input numbers, population sizes, and exponents were used in order to get the best intelligence of GAFIS. The results discovered that the best intelligence of GAFIS was obtained for 5 inputs, the population size of 30, and the exponent of 3. Adding the turbulent kinetic energy as the fifth input the regression value increases from 0.95 to 0.98. This means that considering the turbulent kinetic energy the GAFIS reaches a higher level of intelligence by distinguishing the more difference between the learned data. At the best intelligence, the predicted temperatures by the GAFIS were the same as those predicted by the CFD. The regression number for this condition was around 0.98. Then, using the GAFIS, a correlation was developed to relate the temperature of the node to the inputs (i.e. cylindrical coordinates, inlet temperature, and TKE).

## Roman symbols

C_p_: Specific heat capacity at constant pressure (J/kg K)
d_s_: Nanoparticle diameter (m)
d_water_: Water molecular diameter (m)
k: Thermal conductivity (W/m K)
k_turb_: Turbulent conductivity (W/m K)
*k*: Turbulence kinetic energy (m^2^/s^2^)
*p*: Static pressure (N/m^2^)
Pr: Prandtl number
R: Correlation coefficient or regression number
Re: Reynolds number
T: Temperature (K)
U: Velocity (m/s)

## Greek letters

ε: Dissipation rate of turbulence kinetic energy (m^2^/s^3^)
µ: Dynamic viscosity (kg/m s)
µ_turb_: Turbulent viscosity (kg/m s)
ρ: Density (kg/m^3^)
∅: Particle volume fraction

## Subscriptions

eff: Population size
s: Population size

## Abbreviations

GA: Genetic algorithm
FIS: Fuzzy inference system
TKE: Turbulent kinetic energy
FCM: Fuzzy c-mean
PS: Population size
